# The predictive value of serum concentrations of anti-Müllerian hormone for oocyte quality, fertilization, and implantation

**DOI:** 10.5935/1518-0557.20170035

**Published:** 2017

**Authors:** Edson Borges, Daniela P. A. F. Braga, Amanda Setti, Rita de Cássia Figueira, Assumpto Iaconelli Jr

**Affiliations:** 1 Fertility Medical Group, São Paulo, SP - Brazil; 2 Instituto Sapientiae - Centro de Estudos e Pesquisa em Reprodução Assistida, São Paulo, SP - Brazil; 3 Disciplina de Urologia, Área de Reprodução Humana, Departamento de Cirurgia, Universidade Federal de São Paulo, São Paulo, SP - Brazil

**Keywords:** Oocyte, blastocyst, embryo implantation, pregnancy, COS cells, anti-Müllerian hormone

## Abstract

**Objective:**

This study aimed to identify a possible correlation between serum levels of anti-Müllerian hormone (AMH) and oocyte quality, embryo developmental competence, and implantation potential.

**Methods:**

4488 oocytes obtained from 408 patients undergoing ICSI cycles were evaluated. Oocyte dimorphisms, embryo quality on days two and three, blastocyst formation competence, fertilization rates, implantation rates, and pregnancy rates were correlated with serum levels of AMH using Pearson's correlation coefficient and regression analysis.

**Results:**

A positive correlation was observed between serum levels of AMH and number of retrieved oocytes (CC: 0.600, *p*<0.001), fertilization rate (CC:0.595, *p*=0.048), and number of obtained embryos (CC:0.495, *p*<0.001). AMH did not affect the quality of cleavage stage embryos or the chance of blastocyst formation. However, AMH levels affected oocyte quality (OR:0.75, CI 0.44-0.96, *p*<0.001), and implantation (CC:0,116, *p*=0.031) and pregnancy (OR:1.22, CI:1.03-1.53, *p*<0.001) rates.

**Conclusion:**

Serum levels of AMH are a useful predictor of ovarian response to COS, oocyte quality, and fertilization. However, AMH levels may also compromise clinical outcomes; lower AMH levels did not impair embryo development.

## INTRODUCTION

Anti-Müllerian hormone (AMH), also known as Müllerian inhibiting substance, is a peptide growth factor and a member of the tissue growth factor beta superfamily. In male fetuses, the development of the internal reproductive tract requires the presence of AMH to cause the involution of the Müllerian ducts, the anlagen of the female reproductive tract (Nussey & Whitehead, 2001). In females, fetal AMH is produced after 36 weeks of gestation by the granulosa cells of primary, secondary, pre-antral, and early antral follicles (Durlinger *et al*., 1999; Rajpert-De Meyts *et al*., 1999; Weenen *et al*. 2004), and it continues to be expressed until the follicles reach the antral phase and are more than 2mm in diameter (Messinis, 2006).

It has been suggested that AMH inhibits the initiation of primordial follicle growth from the ovarian reserve (Weenen *et al*., 2004; Nilsson *et al*., 2007). In mice, it decreases the sensitivity of follicles to FSH, thereby inhibiting FSH-induced antral follicle growth (Durlinger *et al*., 2002). Although not fully understood, AMH may play a role in regulating the recruitment of cohorts of antral follicles (reviewed in (Baerwald *et al*., 2012).

The anti-Müllerian hormone, barely detectable at birth in females, peaks after puberty (Rajpert-De Meyts *et al*., 1999). In line with this, AMH levels have shown greater sensitivity to ovarian aging (de Vet *et al*., 2002) and a stronger relationship with the number of early antral follicles (Fanchin *et al*., 2003). In fact, the serum level of AMH has been established as the most valuable marker of ovarian reserve, as it correlates highly with the baseline antral follicle count. In addition, AMH levels are a useful predictor of ovarian response to controlled ovarian stimulation (COS) (van Rooij *et al*., 2002; Eldar-Geva *et al*., 2005; Tremellen *et al*., 2005; Nakhuda *et al*., 2006; Nakhuda *et al*., 2007; Nardo *et al*., 2009).

Previous studies found that, besides ovarian response to COS, the serum level of AMH can be used as a predictor of severity of endometriosis (Shebl *et al*., 2009). Anti-Müllerian hormone measurements also offer relatively high specificity and sensitivity as a diagnostic marker for polycystic ovary syndrome (Pigny *et al*., 2006). Moreover, as AMH declines with age and continues to decline until it reaches a stage in which it becomes undetectable in serum after menopause, it has also been suggested that a single AMH measurement may be a good predictor of the onset of menopause in aging women.

Despite being a good marker of ovarian response, AMH fails to predict the outcome of pregnancy (Lie Fong *et al*., 2008; Talebian *et al*., 2008; Riggs *et al*., 2011), since, as suggested by Loh & Maheshwari (2011), it cannot predict the quality of the oocyte. However, previously published reports have focused on the assessment of embryo quality (Hazout *et al*., 2005; Silberstein *et al*., 2006; Smeenk *et al*., 2007; Lie Fong *et al*., 2008; Talebian *et al*., 2008) rather than oocyte quality, considering specifically intracytoplasmic and extracytoplasmic oocyte defects.

In fact, the number of retrieved oocytes and the quality of oocytes and embryos significantly impact the outcomes of assisted reproductive technology (ART). It has been reported that, although the percentage of embryo transfers that leads to deliveries has steadily increased, in the last years, the vast majority of embryos that are produced *in vitro* fail to develop into an infant (Kovalevsky & Patrizio, 2005). Therefore, the goal of the present study was to identify a possible correlation between serum levels of AMH and the following: (i) oocyte quality, (ii) embryo developmental competence, and (iii) implantation potential.

## MATERIALS AND METHOD

### Study Design

This study analyzed the correlation between serum levels of AMH and (i) maternal age, (ii) maternal BMI, (iii) FSH dose for ovarian stimulation, (iv) number of aspirated follicles, (v) number of retrieved oocytes, (vi) number of retrieved MII mature oocytes, (vii) oocyte quality, (viii) fertilization rate, (ix) number of obtained embryos, (x) number of obtained high-quality embryos, (xi) embryo quality on day two, (xii) embryo quality on day three, (xiii) chance of blastocyst formation, (xiv) number of transferred embryos, (xv) implantation rate, and (xvi) pregnancy rate.

Intra and extracytoplasmic defects were recorded before sperm injection for purposes of oocyte quality assessment. In embryo quality evaluation, the embryos were categorized as high or low quality on days two, three, and five. Fertilization rate was defined based on the number of oocytes presenting two clearly distinct pronuclei 18 hours after ICSI divided by the number of injected oocytes. Implantation rate was calculated as the number of gestational sacs divided by the number of embryos transferred per patient. Clinical pregnancy was defined by the presence of a gestational sac with heartbeat viewed on ultrasound examination 4-6 weeks after embryo transfer.

The assessment included 4488 oocytes obtained from 408 patients undergoing ICSI cycles between January 2011 and August 2013. Serum AMH tests were included as a standard measure in the IVF program. Tests were run prior to the start of each cycle and AMH levels were recorded. The oocytes were evaluated immediately before sperm injection, and the embryos were evaluated 16-18h post-ICSI and on days two, three and five of development.

Inclusion criteria were as follows: women of good physical and mental health, with regular menstrual cycles of 25-35 days, normal basal FSH and LH levels and BMI lower than 30kg/m^2^, presence of both ovaries and an intact uterus, undergoing the first or second ICSI cycle. Patients with endometriosis or gynecological/medical disorders and a negative result in a screening for sexually transmitted diseases were excluded. All cases of severe alteration in spermatogenesis, including frozen and surgically retrieved sperm, were also excluded from the study.

The implantation rate was defined as the total number of gestational sacs divided by the total number of transferred embryos. Clinical pregnancy was defined as the presence of a gestational sac viewed on ultrasound examination 4-6 weeks after embryo transfer.

The patients gave written consent before joining the study and agreed to share the outcomes of their cycles for research purposes. The local review board approved the study.

### Controlled ovarian stimulation

Controlled ovarian stimulation was achieved using a daily dose of recombinant FSH (Gonal-F; Serono, Geneva, Switzerland) starting on day three of the cycle. Pituitary down-regulation was performed using a GnRH antagonist (Cetrotide, Serono, Geneva, Switzerland), started when at least one follicle ≥14mm was viewed.

Follicular growth was monitored using transvaginal ultrasound examination starting on day four of gonadotropin administration. When adequate follicular growth and serum E2 levels were observed, recombinant hCG (Ovidrel; Serono, Geneva, Switzerland) was administered to trigger final follicular maturation. The oocytes were collected 35 hours after hCG administration through transvaginal ultrasound-guided ovum pick-up.

### Preparation of oocytes

The retrieved oocytes were maintained in culture medium (Global^®^ for fertilisation, LifeGlobal, Connecticut, USA) supplemented with 10% protein supplement (LGPS, LifeGlobal, Connecticut, USA) and covered with paraffin oil (Paraffin oil P.G., LifeGlobal, Connecticut, USA) for two to three hours before the removal of cumulus cells. The surrounding cumulus cells were removed after exposure to a HEPES-buffered medium containing hyaluronidase (80IU/mL, LifeGlobal, Connecticut, USA). The remaining cumulus cells were gently removed with a hand-drawn Pasteur pipette (Humagen Fertility Diagnostics, Charlottesville, USA).

Oocyte morphology was assessed immediately before sperm injection (4 hours after retrieval) using an inverted Nikon Diaphot microscope (Eclipse TE 300; Nikon^®^, Tokyo, Japan) with a Hoffmann modulation contrast system under 400X magnification. The following oocyte dysmorphisms were recorded: intra-cytoplasmic defects such as (i) cytoplasm color, (ii) vacuoles in the ooplasm, (iii) aggregates of smooth endoplasmic reticulum clusters (ERC) in the ooplasm and (iv) retractile bodies; and extracytoplasmic defects such as (i) large perivitelline space (PVS), (ii) PVS granularity, (iii) zona pellucida (ZP) abnormalities, (iv) shape abnormalities, and (v) fragmented polar body (PB).

Oocytes releasing the first polar body were considered mature and were used for ICSI.

### Intracytoplasmic sperm injection

Intracytoplasmic sperm injection was performed in a microinjection dish prepared with 4-µL droplets of buffered medium (Global^®^ w/HEPES, LifeGlobal, Connecticut, USA) and covered with paraffin oil on the heated stage of an inverted microscope (37.0±0.5°C). Approximately 16 hours after ICSI, fertilization was confirmed by the presence of two pronuclei and the extrusion of the second polar body. Embryos were maintained in a 50-µL drop of culture medium (Global^®^, LifeGlobal, Connecticut, USA) supplemented with 10% protein supplement and covered with paraffin oil in a humidified atmosphere under 6% CO_2_ at 37°C for three days.

### Embryo morphology evaluation

Embryo morphology was assessed 16-18h post-ICSI and on the mornings of days two, three and five of embryo development using an inverted Nikon Diaphot microscope (Eclipse TE 300; Nikon, Tokyo, Japan) with a Hoffmann modulation contrast system under 400X magnification.

Cleavage stage morphology was assessed based on the following parameters: number of blastomeres, percent fragmentation, variation in blastomere symmetry, presence of multinucleation, and defects in the zona pellucida and cytoplasm. High-quality cleavage stage embryos had to show the following characteristics: 4 cells on day two or 8-10 cells on day three, <15% fragmentation, symmetric blastomeres, absence of multinucleation, colorless cytoplasm with moderate granulation and no inclusions, absence of perivitelline space granularity and absence of zona pellucida dysmorphism. Embryos lacking any of these characteristics were considered to be of low quality.

In blastocyst formation assessment the embryos were given a numerical score from 1 to 6 based on their degree of expansion and hatching statuses as follows: 1, an early blastocyst with a blastocoel that was less than half the volume of the embryo; 2, a blastocyst with a blastocoel that was greater than half the volume of the embryo; 3, a full blastocyst with a blastocoel that completely filled the embryo; 4, an expanded blastocyst; 5, a hatching blastocyst; and 6, a hatched blastocyst. Embryos receiving scores of 3 and greater were considered a full blastocyst.

### Blood collection and Anti-Müllerian hormone analysis

When the patients visited the outpatient department, serum AMH levels were obtained, regardless of the menstrual phase, using a human AMH ELISA Kit (CUSABIO, Wuhan, China).

Blood samples were collected by peripheral venipuncture and centrifuged at 1000×g, 4^o^C, for 15 minutes, within 30 minutes of collection; the plasma portion was removed and assayed.

The human AMH ELISA Kits were used according to supplier instructions. The intra- and inter-assay coefficients of variation for all assays were <10 and 15%, respectively. Limits of detection were: 1ng/mL-75ng/mL.

### Statistical analysis

Cycle and patient characteristics were expressed as the mean ± standard deviation for continuous variables; percentages were used for categorical variables.

Pearson's correlation coefficient was used to evaluate the relationship between serum AMH levels and continuous variables. The results were expressed in the form of correlation coefficients (CC) and *p*-values.

Binary regression analysis was performed to evaluate the influence of AMH levels on pregnancy rates, embryo quality, blastocyst formation, and oocyte defects. These regressions were adjusted for female age, total level of FSH used in controlled ovarian stimulation, and the initial dose of FSH, as these are potentially confounding factors in the association between AMH level and the evaluated variables. The results were expressed as odds ratios (OR), 95% confidence intervals (CI) and *p*-values.

Significance was assigned to events with a *p*-value <0.05. Data analysis was carried out on Minitab Statistical Software (version 14).

## RESULTS

Patient and cycle characteristics were as follows: maternal age: 37.7±5.4 (20-43) years, paternal age: 39.0±6.7 (24-65) years; body mass index (BMI): 24.4±3.9 (14.2-37.2); total dose of FSH administered in ovarian stimulation: 2370.9±855.3 (1900-3450)IU; number of aspirated follicles: 8.0±10.5 (1-20); number of retrieved oocytes: 6.7±7.4 (1-25); number of retrieved mature oocytes: 5.9±6.2 (1-18); fertilization rate: 82.7±20.8% (10-100); embryos obtained: 4.5±5.5 (1-16); high-quality embryos obtained: 1.5±1.1 (1-5); embryos transferred: 1.0±1.8 (1-3); implantation rate (%): 22.0±40.2 (0-100); and pregnancy rate: 28.9%.

The causes of infertility were as follows: male factor (146/408:35.8%), tubal factor (26/408:6.4%), unexplained infertility (29/408:7.1%), polycystic ovary syndrome (PCOS, 25/408:6.1%), female factor (127/408: 31.1%), and others (55/408:13.5%).

As expected, an inverse correlation was found between serum AMH levels and maternal age (CC:-0.279, *p*<0.001). Significant positive correlations were identified between serum AMH levels and number of aspirated follicles (CC: 0.626, *p*<0.001), number of retrieved oocytes (CC:0.600, *p*<0.001), number of mature oocytes (CC:0.588, *p*<0.001), fertilization rate (CC:0.595, *p*=0.048), number of obtained embryos (CC:0.495, *p*<0.001), number of high-quality embryos (CC:0.504, *p*<0.001), number of transferred embryos (CC:0.221, *p*<0.001), and implantation rate (CC:0,116, *p*=0.031) (Table 1).

**Table 1:** Pearson's correlation coefficient between the serum level of Anti-Müllerian hormone and patient and cycle characteristics.

**Serum level of AMH**	**Variable**	**Correlation coefficient**	***p***
	Maternal age	-0.279	**<0.001**
	Maternal BMI	-0.203	0.065
	FSH dose	0.089	0.075
	Number of aspirated follicles	0.626	**<0.001**
	Number of retrieved oocytes	0.600	**<0.001**
	Number of retrieved MII mature oocytes	0.588	**<0.001**
	Fertilization Rate	0.595	**0.048**
	Number of obtained embryos	0.495	**<0.001**
	Number of obtained high-quality embryos	0.504	**<0.001**
	Number of transferred Embryos	0.221	**<0.001**
	Implantation Rate	0.116	**0.031**

AMH: Anti-Müllerian hormone.

Serum levels of AMH also impacted pregnancy rates (OR:1.22, CI:1.03-1.53, *p*<0.001, Table 2).

**Table 2:** Binary regression analysis of the effect of the serum level of Anti-Müllerian hormone on embryo quality on days two or three, chance of blastocyst formation, and pregnancy odds

Predictor variable	Response variable	*P*	OR	CI: Lower	CI: Upper
Serum level of AMH	Embryo quality on day two	0.846	1.01	0.97	1.03
	Embryo quality on day three	0.724	0.99	0.73	2.19
	Blastocyst formation	0.574	0.98	0.54	1.96
	Pregnancy	**<0.001**	1.22	1.03-	1.53

AMH: Anti-Müllerian hormone.

The FSH dose that was used for ovarian stimulation (CC:0.089, *p*=0.075) and female BMI (CC:-0.203, *p*=0.065) did not correlate with serum AMH levels.

The serum level of AMH did not impact embryo quality on days two or three or the chance of blastocyst formation. However, AMH levels affected oocyte quality (OR:0.75, CI:0.44-0.96, *p*<0.001, Table 3).

**Table 3:** Binary regression analysis of oocyte dysmorphisms affected by the serum level of Anti-Müllerian hormone

Predictor variable	Response variable	*p*	OR	CI: Lower	CI: Upper
**Serum level of AMH**	At least one oocyte defect	<0.001	0.75	0.44	0.96
	Cytoplasm color	0.056	0.79	0.65	1.03
	Vacuoles in the ooplasm	0.465	0.97	0.91	1.09
	Aggregates of smooth ERC	**0.025**	0.82	0.63	0.99
	Retractile Bodies	0.256	0.83	0.53	1.53
	Large PVS	**<0.001**	0.84	0.76	0.93
	PVS granularity	**<0.001**	0.91	0.79	0.96
	ZP abnormalities	**<0.001**	0.85	0.73	0.94
	Shape abnormalities.	**0.010**	0.83	0.51	0.93
	Fragmented PB	0.456	0.99	0.86	1.09

AMH: Anti-Müllerian hormone, ERC: Endoplasmic reticulum cluster, PVS: perivitelline space, ZP: Zona pellucida, PB: Polar Body.

The presence of aggregates of smooth ERC (OR:0.82, CI:0.63-0.99, *p*=0.025), large PVS (OR:0.84, CI:0.76-0.93, *p*<0.001), PVS granularity (OR:0.91, CI:0.79-0.96, *p*<0.001), ZP abnormalities (OR:0.85, CI:0.73-0.94, *p*<0.001), and shape abnormalities (OR:0.83, CI:0.51-0.93, *p*=0.010) were affected by the serum level of AMH. The presence of other dysmorphisms, such as cytoplasm color, vacuoles in the ooplasm, retractile bodies, and fragmented PB was not affected by the level of AMH (Table 3).

## DISCUSSION

Oocyte quality has been regarded as a variable that influences the implantation of derived embryos. It has been previously described that when cumulus cells are denuded for ICSI, more than 60% of all oocytes show at least one abnormal morphological characteristic (Braga *et al*., 2013). Considering that both extreme forms of ovarian response (low and hyper-response) may be associated with diminished oocyte quality (van Rooij *et al*., 2003; Figueira *et al*., 2011; Braga *et al*., 2012), we have hypothesized that lower levels of AMH might have a negative effect on oocyte and embryo development. Our results demonstrated that neither embryo quality on the cleavage stage nor the chance of blastocyst formation was correlated with AMH levels. However, the serum level of AMH was positively correlated with oocyte quality.

It remains unclear whether the correlation between AMH and the presence of oocyte defects is due to decreased secretion by granulosa cells in poor quality oocytes or due to a possible detrimental effect of low levels of AMH on oocyte quality. Indeed, AMH might play an important role in primordial follicle selection and cyclic growing follicle recruitment (Carlsson *et al*., 2006). Moreover, AMH might regulate the selection of the dominant follicle through the inhibitory effects of AMH on the initial recruitment of primary follicles from the resting primordial follicle pool (Durlinger *et al*., 2001) and through the regulation of FSH sensitivity in the human ovary (Kevenaar *et al*., 2007).

Our data also support previous reports on the prognostic value of AMH levels on female age (Broer *et al*., 2009; Nelson *et al*., 2011) and ovarian response (Broer *et al*., 2009). However, the inability to estimate embryo quality and blastocyst formation competence by AMH levels disagreed with the work of Silberstein *et al*. (2006), in which a relationship was reported between AMH levels and embryo morphology. In the mentioned study, the morphological assessment of cleavage stage embryos consisted of the evaluation of cell number, percent fragmentation, and blastomere symmetry. The presence of multinucleation and defects in the zona pellucida and cytoplasm was not evaluated. Moreover, it failed to define high/low-quality embryos or to adopt any form of embryo scoring criteria.

In our study, the serum level of AMH affected the presence of intra- and extracytoplasmic defects. This finding agreed with an elegant trial from Fanchin *et al*. (2007), in which a clinical model of monodominant follicle *in vitro* fertilization to determine whether AMH production by a single, preovulatory follicle, as assessed by follicular fluid AMH measurements, was positively related to oocyte and embryo development. A direct link was suggested between the aptitude of granulosa cells to produce AMH and the functional quality of the oocyte, as reflected by its competence to become an embryo with implantation potential.

Ebner *et al*. (2006) also demonstrated that serum levels of AMH were associated with oocyte quality in stimulated cycles. In their study, embryo quality was not estimated using baseline AMH levels, which was also in accordance with our evidence. However, the fertilization rate was not correlated with the level of AMH, which disagreed with the results of the present study.

The process of fertilization is initiated by gamete fusion and can be considered to include all the events associated with egg activation until the time the egg is committed to embryonic development (Nixon *et al*., 2000). The observation that the level of AMH negatively affected fertilization and oocyte quality suggests a possible relationship between granulosa cell metabolism and oocyte developmental competence. Previous studies have shown that the degree of apoptosis of mural and cumulus granulosa cells negatively affects the developmental competence of the oocyte (Nakahara *et al*., 1997; Zeuner *et al*., 2003). Accordingly, human (Weenen *et al*., 2004) and animal (Bézard *et al*., 1987; Baarends *et al*., 1995) follicular atresia prevents the expression of AMH.

In this study, it is unclear why AMH serum levels affected fertilization rates and oocyte quality, but not embryo development. It may be suggested that, although the level of AMH has a significant negative effect on oocyte quality and fertilization capacity, once fertilized, oocytes do not show compromised developmental competence *in vitro*. Embryo genome is known to begin express between the four- and eight-cell stages of human embryo development. At this stage, the sperm-derived genes linked to embryo viability have also been disrupted (Tesarík *et al*., 1988).

In addition to fertilization, our results showed the existence of a relationship between level of AMH and chance of pregnancy. Loh & Maheshwari (2011) previously described that AMH fails to predict the odds of pregnancy. Nonetheless, some reported that extremely low levels of AMH are associated with non-pregnancy (La Marca *et al*., 2011) while others described moderate to reasonable pregnancy rates following extremely low levels of serum AMH (Lamazou *et al*., 2011; Weghofer *et al*., 2011). In our study, the chance of pregnancy and the number of obtained embryos, high-quality embryos, and transferred embryos were positively correlated with the level of AMH. Therefore, it might be argued that the correlation between AMH and pregnancy depends on the number of obtained oocytes and embryos available for transfer, rather than embryo quality. On the other hand, AMH levels also negatively impacted the implantation rate, which measures the number of transferred embryos.

These findings raised the question as to whether the worse outcomes observed for patients with lower AMH levels correlated with lower response to COS, decreased oocyte quality or both.

In conclusion, our findings indicated that the serum level of AMH is a useful predictor of ovarian response to COS, oocyte quality, fertilization, and implantation. However, although AMH levels might compromise pregnancy outcomes, lower levels of AMH do not impair the embryo developmental competence.
